# Impending Flop for Brand Antiretrovirals in the Emerging Markets?

**DOI:** 10.2174/1874613600802010068

**Published:** 2008-08-16

**Authors:** Dionisio Daniele, Messeri Daniela

**Affiliations:** 2 “Access to Drugs: International Policies” - CLIA (Italian Network for International Fight against AIDS), Italian Society for Infectious and Tropical Diseases (SIMIT), Division of Infectious Diseases, Pistoia Hospital, Pistoia, Italy; 2 Division of Infectious Diseases, Pistoia Hospital, Pistoia, Italy

## Abstract

Forecasts from Country choices, South-South partnerships and Clinton Foundation-UNITAID coalition show that present policies for brand ARVs are at the risk of flop in emerging South markets such as India, China, Thailand and Brazil.

The dynamics explored in this article highlight the risks the originator companies are running in the emerging markets, along with their interest in direct agreements with the generic industry for the manufacturing and marketing of ARVs.

Resulting information here would suggest the brand enterprises:

- To look for fast registration of their ARVs by regulatory authorities in all countries enlisted for differential pricing.
- To secure all formulations differentiated prices.
- To align with the Clinton-UNITAID prices for the corresponding generics.
- To pursue flexible negotiations with the generic companies to secure both counterparts long-term advantages.

## INTRODUCTION

Generic companies are supplying the developing countries (sometimes through Compulsory Licensing-CL) with cheap copies of brand antiretroviral drugs (ARVs) for HIV infection [1, 2]. Brand companies, in turn, have marketed all newer ARVs, stipulated Voluntary Licenses-VLs with generic firms, pursued differential pricing, and not enforced patents in some cases.

Unfortunately, newer drugs are under patent in India and other supplier countries (India applied on 1^st^ January 2005 the Trade-related Aspects of Intellectual Property-TRIPS rules of the World Trade Organization-WTO) [1]; moreover, CL has resulted in pressure from brand industries and wealthy countries, VLs only account for small fraction procurement, while differential pricing has failed to meet the promised coverage due to sometimes delayed drug registration in entitled countries [1, 2]. Eventually, non-enforcement policies have been implemented at the brand company discretion only [2].

As a result, unaffordable prices still bar the end-users in the resource-poor countries from accessing these lifesaving drugs. Under this backcloth, prospects from Country choices, South-South partnerships and Clinton-UNITAID coalition show that current trade policies for brand ARVs by private pharmaceutical sector are at the risk of flop in emerging markets in the South [3].

## COUNTRY CHOICES

Section 3 (d) of the Indian Patent Act denies patentability to “a new form of a known substance” unless it results in “enhancement of the known efficacy” [4]. Coherently, India’s Patent Office could reject the pending Abbott’s patent application for heat stable lopinavir/r (LPV/r). If so, the Indian firms would be allowed to continue manufacturing the corresponding copy. Noteworthy, the same Office very recently refused as “evergreening” a Boehringer-Ingelheim’s patent application for nevirapine (NVP) syrup.

These insights take into account the recent withdrawal by Glaxo SmithKline and Novartis of patent applications for ARVs in India, after the Indian Court rejected (August 2007) a Novartis challenge to the mentioned Section of Law [5, 6]. In the meanwhile, the US Patent Office rejection (January 2008) of already enforced Gilead’s tenofovir (TDF) patents in USA will likely compel the company to withdraw patent application in India based on predictable rejection by the Indian Patent Office too [7].

This scenario includes the currently working Indian AIDS Control Organization’s (NACO) plan to provide 5,000 first-line resistant people with second-line Indian generic ARVs for free [8].

Why, in such a context, should the Indian government grant the multinationals their patent applications, so disregarding substantial home interests?

China currently produces first-line ARVs along with the active pharmaceutical ingredients (APIs) for second lines too: really, China could become the ARV factory for the poor world because of its cheapest APIs and industrial scale-up very interested in the under-served markets. Unequivocal choices are lacking, however, between pursuance of TRIPS flexibilities of WTO (which China belongs to) [1] and huge business with the multinational giants (including direct price cuttings for brand ARVs).

To date, the government has snobbed the Indian ARVs even though they are cheapest through the Clinton Foundation, a consortium which China is a member of. How much longer will it be worthwhile to Chinese government ignoring the saving money opportunities by Indian generics? Not for long, if China-India trade agreements of November 2006 and January 2008 are supposed to give rise to a mutually profitable partnership for ARVs manufacturing and marketing.

The USA-India strategic partnership, boosted by the “civil nuclear power” agreement of March 2006 [9], has resulted in exploitation of Indian ARVs inside the President’s Emergency Plan for AIDS Relief (PEPFAR), with more than 52 Indian generic ARVs approved to date by the Food and Drug Administration (FDA). In such a perspective it may be worth underscoring that US Patent Office rejection of Gilead’s TDF patents, as mentioned before, came just after FDA had approved Indian Matrix copy for use in PEPFAR [10]: do the strategy balances in South-East Asia weigh now more than brand drug patent defence inside the partnership with India?

In October 2007, Canada notified WTO a CL authorising Canadian manufacturer Apotex to produce WHO (World Health Organization) pre-qualified fixed-dose combination (FDC) copies of three patented ARVs for export to Rwanda: this was the first time a generic firm in the developed world engaged to secure ARVs an African country [11].

Brazil imports brand ARVs at the lowest prices, but the steadily enhanced purchase has more than doubled the expenditure, indicating that the costs for ARVs will continue to climb [12]. The country still purchases heat stable LPV/r from Abbott at US$1000 person/year: definitely a too expensive price comparing to an Indian copy available through the Clinton Foundation (which Brazil belongs to) at $695!

Why shouldn’t Brazil take advantage of Clinton opportunities? Possibly it is ready, while only waiting for rejection of Abbott’s patent application for heat stable LPV/r in India. This couples with a Ministry of Health’s decree on 9 April 2008 signalling that Brazil might reject patent request for Gilead’s TDF (due to its high cost), and import a generic copy [13].

Should these prospects come true, Abbott and Gilead would lose Brazilian HIV/AIDS market.

Following past government’s CL policy against Merck’s efavirenz (EFV) and Abbott’s LPV/r, Thailand imports EFV and heat stable LPV/r as generics from India and has planned for domestic manufacturing: in the meantime, it has been placed on the USA Priority Watch List [14]. Would Thailand be up to resisting? It looks like it would be because: 1) the country can import ARVs from Indian manufacturers or the Clinton Foundation (whose consortium is part), 2) it can rely on favourable international balances, while enjoying the advantages from South-South partnerships, 3) the new coalition government, started on February 2008, looks like it would be prone to go on with CL policy [15]. If these forecasts were fulfilled, the brand companies should give up Thailand.

## SOUTH-SOUTH PARTNERSHIPS

**Figures (1):**
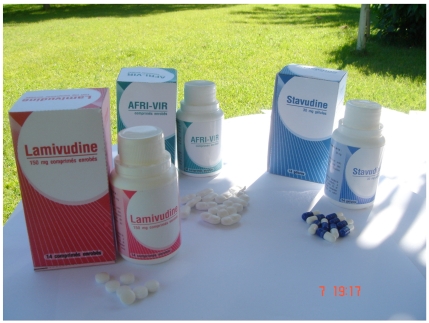
Generic ARVS by an African company (Pharmakina, Democratic Republic of Congo).

Partnerships (between country governments or generic drug companies) for building ARVs plants are mushrooming in Africa, where they fall into the African Union and the Economic Community of West African States (ECOWAS) self-sufficiency plans (Fig. **1**) [16, 17]. These partnerships might, in the future, undermine markets to the brand corporations, while pushing them into flexible negotiations with the generic competitors. Actually, balanced VL agreements for the manufacturing of ARVs have already been signed: i.e. the Bristol-Myers Squibb (BMS) and Tibotec Pharmaceuticals agreements with Aspen Pharmacare of South Africa, and the Roche agreements with Addis Pharmaceutical Factory of Ethiopia and Varichem Pharmaceuticals of Zimbabwe [18-20].

These opportunities are spurring the generic manufacturers into innovation while insisting on VL deals with the brand industry to exploit know-how, training and technology transfer opportunities for developing new ARVs and gaining the wealthy markets.

## CLINTON-UNITAID COALITION

Clinton Foundation’s HIV/AIDS Initiative (CHAI) is lowering more and more the prices of generic ARVs by partnering with UNITAID.

As International Drug Purchase Facility primarily financed from the proceeds of a tax levied on airline tickets, UNITAID is established to provide long-term funding to increase access to drugs and diagnostics for HIV/AIDS, malaria and tuberculosis in the resource-poor countries [21, 22].

Concurrently, lists of countries on differential pricing have been arranged by Abbott, Boehringer-Ingelheim, Bristol-Myers Squibb, Gilead, GlaxoSmithKline, Merck &amp; Co. Inc, and Roche enterprises: regrettably, their prices are (with isolated exceptions) quite higher than the corresponding CHAI generics [23], with a perceivable risk by considering that:

- Some FDC ARVs, still produced by generic firms only (Table **1**) [23], have been made available to the South markets just thanks to CHAI-UNITAID discounts.
- Further countries are expected to join the presently 73 Clinton Foundation members, so slashing prices due to enhanced bulk procurement of ARVs.

## TACKLING EVOLUTIONARY TRENDS

The dynamics analysed in this paper have highlighted the risks (bound up with their ARVs policies) the originator companies are running in the South markets, while remarking, at the same time, their interest in VL agreements with the generic industry for the manufacturing and marketing of ARVs. Cross information here would suggest the brand enterprises:

- To look for fast registration of their ARVs by regulatory authorities in all countries enlisted for differential pricing.
- To secure all formulations differentiated prices.
- To align with the prices of corresponding CHAI generics (to enhance competition, based on CHAI is oriented towards WHO and /or FDA approved drugs).
- To pursue flexible negotiations with the generic companies to secure both counterparts long-term advantages.

## CONTRIBUTORSHIP

- D. Dionisio conceived, designed and wrote the article.
- D. Messeri shared in the draft preparation and participated in overall interpretation, revision and harmonisation.

## Figures and Tables

**Table 1.** Arv Drug Combinations Only Available as Generics

**AZT/3TC/NVP**: Adult formulations by Aspen, Apotex, Aurobindo, Cipla, Hetero, Matrix, Emcure, Ranbaxy, and Strides. The Clinton Foundation has negotiated reduced prices with Cipla and Matrix. Paediatric formulations by Government Pharmaceutical Organization (GPO) and Ranbaxy.
**D4T/3TC/NVP**: The Clinton Foundation has negotiated with Hetero, Matrix, Cipla and Ranbaxy reduced prices for adult formulations. Adult formulation also made by Emcure and Strides. Paediatric formulations by Cipla, GPO, Emcure, and Ranbaxy.
**D4T/3TC**: Adult formulations by Aurobindo, Cipla, Hetero, Matrix, Ranbaxy, Emcure, and Strides. Paediatric formulations by Emcure and Ranbaxy.
**D4T/3TC+EFV**: Adult formulations by Cipla, Emcure, Strides and Ranbaxy. No paediatric formulations.
** AZT/3TC+EFV**: Adult formulations by Aurobindo, Cipla, Emcure, Ranbaxy, and Strides. No paediatric formulations.
**PMTCT:NVP+AZT**:Granule formulations by Strides.
** TDF/3TC**:Adult formulation by Matrix (reduced price in the Clinton Foundation’s consortium). Not for paediatric use.
** TDF/3TC+EFV**:Adult formulation by Cipla. Not for paediatric use.
**TDF/3TC/EFV**:Adult formulation by Matrix (reduced price in the Clinton Foundation’s consortium). Not for paediatric use.

AZT=zidovudine, 3TC=lamivudine, NVP=nevirapine, D4T=stavudine, EFV=efavirenz, TDF=tenofovir, PMTCT=prevention mother-to-child transmission. WHO prequalified ARVs (updated list) at http://healthtech.who.int/pq/status/Product Registry.aspx?list=ha
